# The expression of Lin28a in the ovaries and the association of *Lin28a* and *Lin28b* with litter size in goats

**DOI:** 10.5194/aab-68-435-2025

**Published:** 2025-07-01

**Authors:** Huili Liang, Xingyu Du, Yujing Xie, Mingxing Chu, Guiling Cao

**Affiliations:** 1 School of Agriculture and Biology, Liaocheng University, Liaocheng 252059, China; 2 State Key Laboratory of Animal Biotech Breeding, Institute of Animal Science, Chinese Academy of Agricultural Sciences (CAAS), Beijing 100193, China

## Abstract

*Lin28a* and *Lin28b* serve as crucial regulators in gametogenesis. However, it remains unclear whether the two genes are expressed in goat ovarian tissues. In the present study, we examined the distribution of Lin28a proteins in the ovarian tissues of juvenile and pubertal Jining Grey (JG) goats. Additionally, we investigated the mutations in *Lin28a* and *Lin28b* and their association with litter size in JG goats. Lin28a proteins were predominantly expressed in the oocytes and granulosa cells of the follicles at different stages of development. Lin28a was also expressed in the corpus luteum, and its expression level declined as the corpus luteum formed and degenerated into the corpus albicans. A mutation (12298T>C) was identified in intron 2 of *Lin28a*, and there was no significant difference ( P>0.05 ) in terms of the kidding number among the three genotypes of JG goats. For *Lin28b*, a mutation (718A>T) in exon 4 was found, presenting three genotypes. The frequency of genotypes AA, AT, and TT was 0.466, 0.504, and 0.03, respectively. The A allele was the dominant allele in JG goats, with an allele frequency of 0.718. At this locus, JG female goats with genotypes TT and AT had 0.83 ( P<0.01 ) and 0.48 ( P<0.05 ) more kids than the goats with genotype AA, and no significant difference ( P>0.05 ) was observed in terms of litter size between the TT and AT genotypes. The present study preliminarily indicated an association between the T allele at the 718 locus in *Lin28b* and a larger litter size in JG goats. Moreover, Lin28a was widely expressed in follicles. These results suggest a potential DNA marker for improving the kidding number in goats and hold significance for goat breeding.

## 1 Introduction

*Lin28a* and *Lin28b* are the two homologs in the mammalian genome of the nematode *Lin28* gene, which was initially identified through a mutant of the nematode *Caenorhabditis elegans* (Ambros and Horvitz, 1984; Moss et al., 1997). Lin28a and Lin28b proteins both contain two highly conserved RNA-binding domains, namely an N-terminal cold-shock domain (CSD) and a C-terminal domain (ZKD) composed of two zinc fingers in tandem with the sequence Cys–Cys–His–Cys (CCHC). Additionally, Lin28b contains a nuclear localization signal (NLS) at its C terminus and a nucleolar localization signal (NoLS) (Cotino-Nájera et al., 2024). *Lin28a* and *Lin28b* regulate many biological processes, including tissue development, cellular growth, pluripotency, and cancer development, through disturbance of the maturation process of *let-7* miRNA or by influencing mRNA translation (Graf et al., 2013; Ustianenko et al., 2018). *Lin28a* and *Lin28b* are mainly expressed in the undifferentiated tissues of the embryo at early stages of development, and the expression abundance decreases gradually with development (Vogt et al., 2012; Faas et al., 2013; Ouchi et al., 2014). *Lin28a* and *Lin28b* also participate in the regulation of somatic reprogramming (Farzaneh et al., 2017; Ding et al., 2022), and abnormal expression causes cancer, including ovarian cancer (Maklad et al., 2023; Zhang et al., 2023a). The cellular localization of the two homologs is different. Lin28b can be localized throughout the cells, even inside the nucleolus, while Lin28a is predominantly localized in the cytosol (Lin et al., 2022), indicating different functions between the two proteins. More and more studies have focused on the roles of *Lin28a* and *Lin28b* in reproduction since they were discovered to be involved in the onset of mammalian puberty.

Previous studies reported that *Lin28a* was expressed in the normal human ovarian surface epithelium (Viswanathan et al., 2009), the ovarian epithelium with no mature follicles and oocytes (Virant-Klun et al., 2011), and the mature human MII oocytes (Assou et al., 2009). In the human fetal ovary, the *Lin28a* transcript level was highest when the gonad contained only primordial germ cells (PGCs) and decreased significantly with increasing gestation, and expression was restricted to primordial and pre-meiotic germ cells during later gestation (Childs et al., 2012; El-Khairi et al., 2012). Mouse Lin28a protein was visible within oocytes and granulosa cells of early-developing follicles, and protein levels appeared to decrease as follicles developed (Grieco et al., 2013). The translation of mRNA plays a crucial role in gametic homeostasis and development and is regulated by RNA-binding proteins. As RNA-binding proteins, Lin28a and Lin28b were also the key regulators of gametogenesis (Gross-Thebing and Raz, 2020). Lin28a and Lin28b regulated PGC formation by inhibiting *let-7* miRNA maturation to promote the expression of *BLIMP1* in mice and chickens (West et al., 2009; Zuo et al., 2021; Suzuki et al., 2023). *Lin28a*-knockout mice showed reduced primordial ovarian follicles (West et al., 2009; Shinoda et al., 2013), and *Lin28b* had a similar effect (Matzuk, 2009; West et al., 2009). However, Flemr et al. (2014) reported a different result. The *Lin28a* and *Lin28b* oocyte-specific knockdown female mice were fertile and produced healthy offspring (Flemr et al., 2014). These discoveries indicate that *Lin28a* and *Lin28b* participate in the self-renewal and/or differentiation of primitive germ cells.

*Lin28a* and *Lin28b* also participate in follicular development. In mice embryonic stem cells, *Lin28a* negatively regulated the oocyte-specific homeobox (*Obox*) genes, which played important roles in the early development of follicles (Rajkovic et al., 2002; Park et al., 2012). In the pig oocytes, reduction of global nucleic acid m^6^A methylation was induced by cycloleucine-impaired oocyte meiosis and subsequent embryo development, possibly through down-regulating *Lin28a* mRNA abundance and disturbing maturation-promoting factor-regulated chromosome and/or spindle organization (Wang et al., 2018). *Let-7a* and *let-7b* were two of the most abundant miRNAs and were widely involved in follicular development, especially atresia (McBride et al., 2012; Gong et al., 2020). *Lin28a* overexpression in the immortal human granulosa cells (HGrC1) decreased the estrogen level, adenosine triphosphate (ATP) content, mitochondrial membrane potential, and glutathione level by suppressing *LARS2* expression to cause mitochondrial dysfunction in granulosa cells (Chen and Liu, 2023). In vitro, *Lin28a* and *let-7b* participated in the regulation of melatonin on the follicular granulosa cells through apoptosis and hormone secretion, and Lin28a protein and melatonin receptor MT-1 protein were co-expressed in the nucleus and the cytoplasm of granulosa cells of sheep ovaries (Zhang et al., 2022, 2023b). The level of *let-7a*, *let-7b*, *let-7c*, and *let-7e* increased in the rat follicular granulosa cells treated with follicle-stimulating hormone (FSH) (Yao et al., 2010). So, *Lin28a* and *Lin28b* and *let-7* miRNA participate in mammalian follicle development. Besides mammals, *Lin28* also regulates the oogenesis in fruit flies. The Lin28 null mutant female fruit flies displayed abnormal late egg chambers and reduced fecundity due to defects in egg chamber formation during early oogenesis, which was the combined result of impaired germline stem cell differentiation and follicle stem cell differentiation (Stratoulias et al., 2014).

The results of the studies mentioned above suggest that *Lin28a* and *Lin28b* have important roles in the ovaries or in follicle development. Whether *Lin28a* and/or *Lin28b* affect goat follicle development or litter size remains unclear. To understand the expression of *Lin28a* and *Lin28b* in goat ovaries and the relationship with litter size in goats, the protein distribution of Lin28a in the goat ovarian tissues was detected using the immunohistochemistry (IHC) method. The polymorphisms of *Lin28a* and *Lin28b* were detected, and the association with litter size in Jining Grey (JG) goats was also investigated. The JG goat breed is a unique local breed in China, with significant characteristics of sexual precocity, year-round estrus, and high prolificity.

## 2 Material and methods

### 2.1 Animals

Clinically healthy female Jining Grey (JG) goats were housed in a goat enclosure and fed ad libitum; 1-month-old ( n=3 ; body weight: 4.43 ± 0.63 kg) and pubertal (2–3 months old; n=3 ; body weight: 7.82 ± 0.78 kg) female JG goats were slaughtered after anesthesia via an injection of 0.1 mL xylazine hydrochloride (Muhua, no. 150804) to collect the ovarian tissues. A total of 236 adult, female, physically mature JG goats which had records of parity and litter size were selected for genotype detection. All the goats were housed at a Jining Grey goat elite reservation farm in Jining City, Shandong Province, China.

### 2.2 Ovarian tissue collection and immunohistochemistry

The ovarian tissues were collected and stored in 4 % paraformaldehyde solution for IHC and kept at 4 °C . Immunohistochemistry was performed according to the manufacturer's instructions for the Rabbit Primary Histostain-Plus IHC Kit (Invitrogen, no. 85-6743). Briefly, samples fixed by paraformaldehyde and paraffin-embedded were separated into paraffin sections. Antigen retrieval was performed by pressure cooking in a 0.01 M sodium citrate buffer (pH 6.0) for 2 min, followed by incubation in 3 % ( v/v ) hydrogen peroxide in methanol for 30 min to block endogenous peroxidase activity; subsequently, sample sections were incubated for 20 min in a blocking buffer. Sections were incubated overnight at 4 °C in a humidified chamber using the following primary antibody: rabbit polyclonal anti-Lin28a antibody ( 1:200 dilution) (Abcam, no. ab83400). Primary antibodies were detected using a goat anti-rabbit secondary antibody and incubation with avidin–biotin–HRP complex (where HRP denotes horseradish peroxidase). Bound antibodies were visualized using 3,3-diaminobenzidine tetrahydrochloride. Slides were counter-stained with hematoxylin, dehydrated using graded alcohols and xylene, and permanently mounted using PerTex (Histolab). Negative controls in which the primary antibody was omitted were included in each experiment. A minimum of four sections per tissue were examined.

### 2.3 Blood sample collection and genomic DNA isolation

Blood samples (5 mL, jugular vein, acid-citrate-dextrose (ACD) anticoagulant) were collected from the 236 JG goats. Genomic DNA was extracted from whole blood sample using phenol–chloroform and was dissolved in a TE buffer (10 $\mathrm{mmol}\;L^{-1}$ Tris-HCl (pH 8.0), 1 $\mathrm{mmol}\;L^{-1}$ EDTA (pH 8.0)). The quality and purity of each DNA sample were measured by means of a NanoDrop 1000 (Thermo Fisher Scientific).

### 2.4 Primer design and identification of candidate mutations

For the *Lin28a* gene, two pairs of primer (G28a1 and G28a2) were designed to amplify the sequence of exon 2 and exon 3 (Table 1 and Fig. S1 in the Supplement) according to the goat *Lin28a* genomic DNA sequence (NC_030809) using OLIGO 6.0. Genomic DNA from 10 JG goats was selected randomly and pooled together as a template for PCR. The PCR products were sequenced from two directions. The sequences and sequencing peak plots were used to search base pair variations. For the *Lin28b* gene, the primer B28E4 was designed to amplify the sequence of exon 4 (Table 1). The genomic DNA pool was also used as a template for PCR and to scan mutation. The primer B718 was used to amplify the sequence around the mutation site found in the fragment of B28E4.

**Table 1:** Primer information.

Primer	Primer sequence ( $5^{\prime }$ to $3^{\prime }$ )	Amplified regions	Product	Annealing
name			size	temperature
G28a1	F: TGAAAGAGAGGTGGTGAAGTG	Partial region of intron 1 and exon 2 of *Lin28a*	693 bp	58 °C
	R: AAATCCCAACAGCCGAGT			
G28a2	F: GGCACTGTCTTACTTCGTAGG	Partial region of intron 2, exon 3, and partial region of intron 3	730 bp	56 °C
	R: CATACATACCACCCTCTCTTTG	of *Lin28a* / *Pma*C I		
B28E4	F: GAAGTACTTCCTTGCCTTATGT	Partial region of exon 4 of *Lin28b*	749 bp	55 °C
	R: GCTATGCATCAACTATGAGGT			
B718	F: GTCAGCTCTCCTTTTTTAATTT	Partial region of exon 4 of *Lin28b* and *Pst* I	146 bp	53 °C
	R: AACTATGAGGTAGCCTTCTG			

### 2.5 Polymorphism detection of *Lin28a* and *Lin28b*

The polymorphisms in *Lin28a* and *Lin28b* can all be analyzed by means of PCR-RFLP (restriction fragment length polymorphism) and CRS-RFLP (create restriction site combined with restriction fragment length polymorphism), performed by mixing 5 µL of PCR product (with primer G28a2 and B718), 5 U (where “U” represents units) of the restriction enzyme *Pma*C I (CA *C* GTG for G28a2 PCR fragment) (Takara, no. 1177) or *Pst* I (C *A* GCAA, for B718 PCR fragment) (Takara, no. 1073), and 1 µL of the corresponding 10× reaction buffer, followed by incubation at the reaction temperature for 4 h and then separation at 120 V on a 1.5 % agarose gel or a 12 % polyacrylamide gel ( acrylamide:bis-acrylamide=39:1 ). The Gel Doc XR System (BioRad) was used to analyze and count genotypes.

### 2.6 Statistical analysis

Allele frequencies, genotype frequencies, P values, polymorphism information content (PIC), heterozygosity (He), and the number of effective alleles (Ne) were calculated using the data obtained from genotyping results. The distribution of genotypes was analyzed according to the Hardy–Weinberg equilibrium using the Chi-square test. Polymorphism information content (PIC) was calculated using Nei's method, implemented in the GDIcall Online Calculator (http://www.msrcall.com/Gdicall.aspx, last access: 30 November 2023) (Czarnik et al., 2009). The following fixed-effect model was employed for the analysis of litter size in JG goat does, and least-squares means were used for multiple comparisons of litter size among the different genotypes.

$$y_{ijklm}=\mu +S_{i}+P_{k}+G_{l}+e_{ijklm}$$

In the above, $y_{ijklm}$ is the phenotypic value of litter size, μ is the population mean, $S_{i}$ is the fixed effect of the i th sire ( i=1 , 2, 3, …, 10), $P_{k}$ is the fixed effect of the k th parity ( k=1 , 2), $G_{l}$ is the fixed effect of the l th genotype ( l=1 , 2, 3), and $e_{ijklm}$ is the random residual effect of each observation.

Analysis was performed using the general linear model procedure of SAS (Ver 9.4) (SAS Institute Inc., Cary, NC, USA). Mean separation procedures were performed using a least significant difference test.

## 3 Results

### 3.1 Distribution of Lin28a protein in goat ovarian tissues

**Figure 1:**
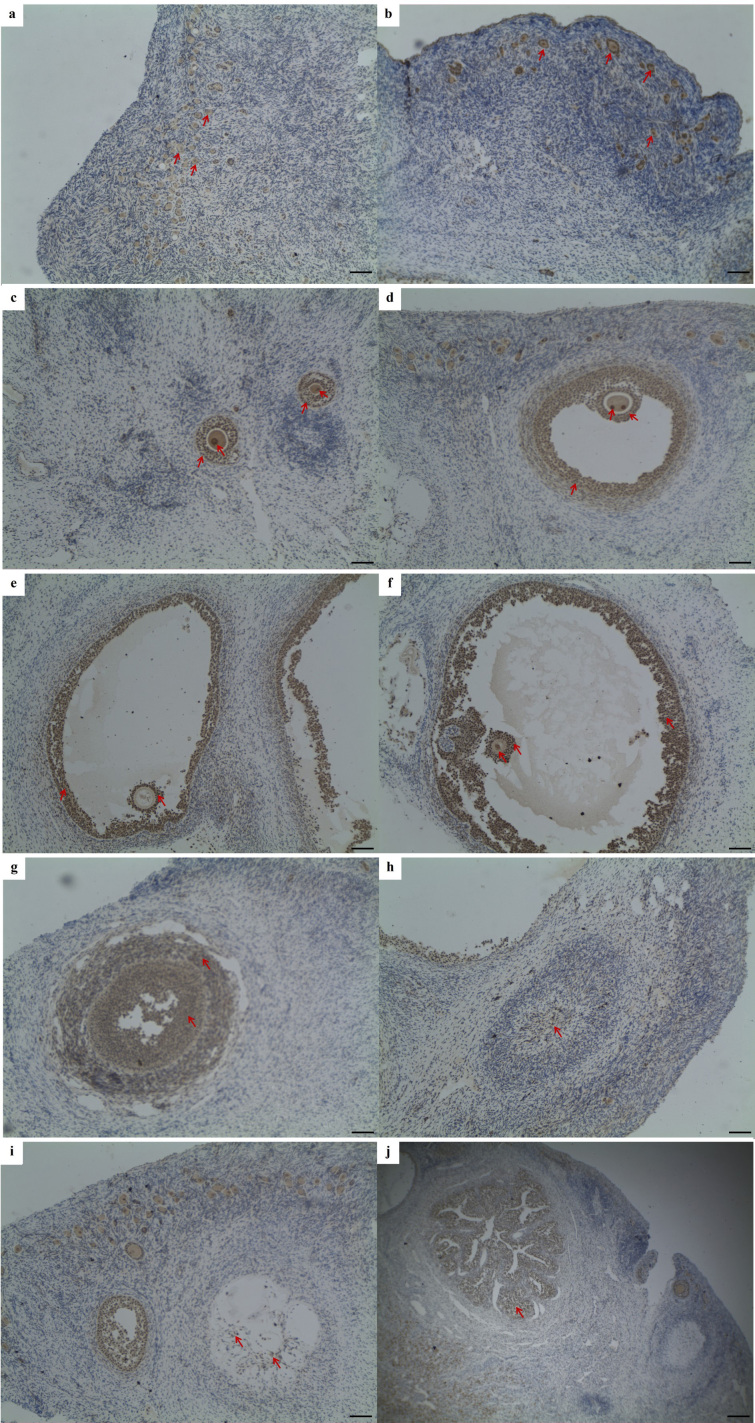
Immunohistochemical staining of Lin28a protein in ovarian tissue ( ×100 ). The Lin28a protein was expressed in ovarian primordial follicles **(a, b)**; in the granulosa cells and oocytes in pre-antral follicles **(c)**; and in the thecal cells, granulosa cells, cumulus cells, and oocytes in antral follicles **(d–f)**. Lin28a protein was also distributed in the granulosa lutein cells and theca lutein cells of the corpus luteum at different stages of development **(g–i)** and in the interstitial glands of the ovaries, developed from atretic follicles **(j)**. Scale bar: 100 µm .

Using the IHC method, Lin28a protein distribution was examined in goat ovarian tissues (Fig. 1). Lin28a-positive cells were concentrated at the ovarian cortex in juvenile goats, mainly in primordial follicles (Fig. 1a). In the ovaries of pubertal goats, Lin28a proteins were distributed in the primordial follicles (Fig. 1b) in the cortical stroma of ovaries and the follicles at different stages of development (Fig. 1c–f). In detail, Lin28a staining was found in the granulosa cells and oocytes of the primary follicles (Fig. 1c). In the secondary follicles and mature follicles, Lin28a was expressed in the theca cells, granulosa cells, cumulus cells, and oocytes in antral follicles (Fig. 1d–f). Lin28a was also found to be expressed in granulosa lutein cells and theca lutein cells of the corpus luteum at different stages of development, and the expression level of Lin28a decreased with the formation of corpus luteum and degeneration to corpus albicans (Fig. 1g–i). Lin28a-positive cells were also observed in the interstitial glands (Fig. 1j). Unfortunately, the lack of suitable antibodies led to unsuccessful IHC of Lin28b in the ovarian tissues.

**Table 2:** Genotype frequencies and population indexes for 12298T>C and 718A>T in JG goats.

Loci	Genotypes	Frequency		He	PIC	Ne	H–W test $\chi^{2}$
	(size)	Genotypes	Alleles				( P value)
12298T>C	TT (86)	0.374	0.596 (T)	0.482	0.366	1.929	1.45
(*Lin28a*)	TC (102)	0.443	0.404 (C)				( P=0.485 )
( n=230 )	CC (42)	0.183					
718A>T	AA (110)	0.466	0.718 (A)	0.387	0.312	1.632	14.26
(Lin28b)	AT (119)	0.504	0.282 (T)				( P=0.001 )
( n=236 )	TT (7)	0.030					

The numbers in the brackets are the genotype individuals.

### 3.2 Polymorphism identification and detection

**Figure 2:**
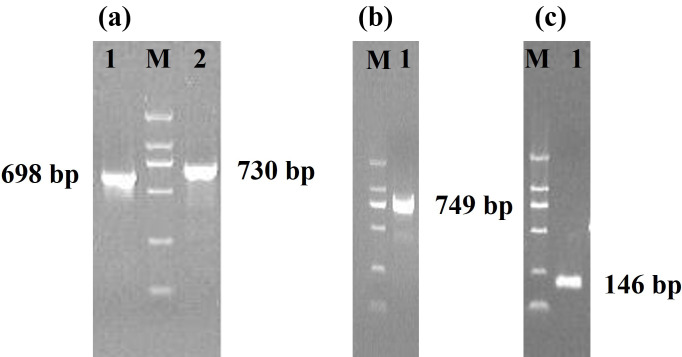
Agarose gel electrophoresis of PCR products. M, DL 2000 DNA marker. **(a)** The PCR products of the primers G28a1 and G28a2. **(b)** The PCR products of the primer B28E4. **(c)** The PCR products of the primer B718.

Three pairs of primers were designed to obtain the sequences of *Lin28a* and *Lin28b* (Table 1). For the *Lin28a* gene, a 698 bp sequence fragment and a 730 bp fragment were obtained with the primers G28a1 and G28a2, respectively (Fig. 2a and Sequences in the Supplement). For *Lin28b*, a 749 bp sequence fragment was obtained with the primer B28E4 (Fig. 2b and Sequences in the Supplement).

**Figure 3:**
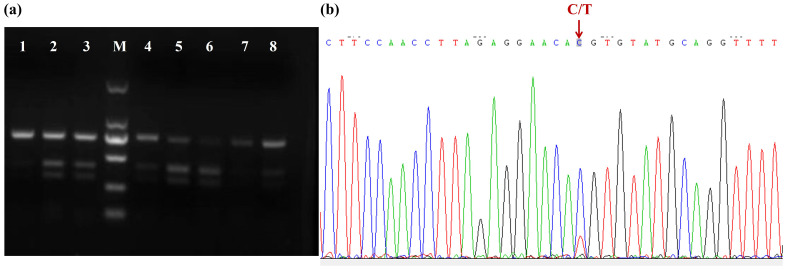
Agarose gel electrophoresis for the PCR-RFLP products and DNA sequence near mutation 12298T>C. **(a)** Agarose gel electrophoresis for the PCR-RFLP products of the amplified fragment of primer G28a2. Lanes 1, 7, and 8: TT; lanes 2, 3, 4, and 5: TC; lane 6: CC. M: D2000 DNA ladder. **(b)** DNA sequence near mutation 12298T>C.

No base variation was found in the 698 bp sequence of *Lin28a* – that is, there was no mutation in exon 2 of *Lin28a*. A base mutation was found in the G28a2 (730 bp) fragment and was named 12298T>C, located in intron 2 of *Lin28a* according to the sequence NC_030809, and no base variation was found in exon 3. The 12298T>C mutation was detected by PCR-RFLP in JG goats (Fig. 3a). As shown in Fig. 3a, the T-to-C transition at the 12298 locus expressed three genotypes: TT, TC, and CC. The sequence of the heterozygous genotype is presented in Fig. 3b.

**Figure 4:**
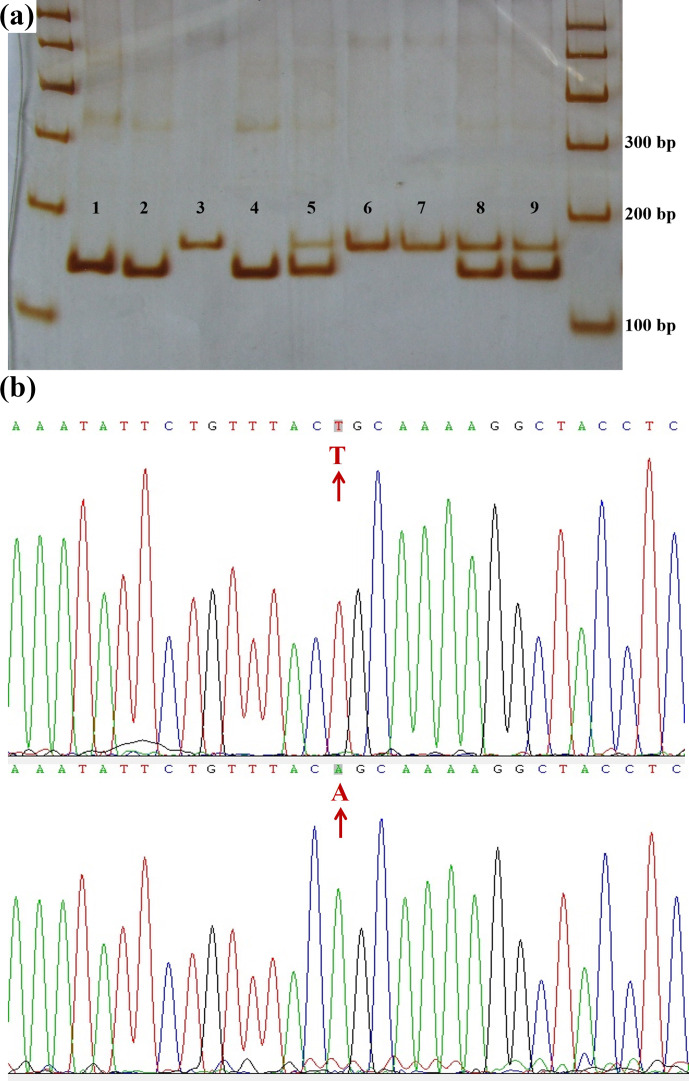
Polyacrylamide gel electrophoresis for the CRS-PCR-RFLP products and DNA sequences near 718A>T. **(a)** Polyacrylamide gel electrophoresis for the CRS-PCR-RFLP products of the amplified fragment of primer B718. Lanes 1, 2, and 4: AA; lanes 3, 6, and 7: TT; lanes 5, 8, and 9: AT. DNA marker: marker I DNA Ladder. **(b)** DNA sequence near the mutation 718A>T.

In the *Lin28b* gene, a base transversion was found in the 749 bp sequence and was named 718A>T. The sequence around the 718 site (Ca/tGCAA) was similar to the recognition sequence (C *t* GCAG) of the restriction enzyme *Pst* I. So, the CRS-RFLP method was used to detect the genotypes of JG goats (Sequences in the Supplement). A 146 bp fragment (Fig. 2c) containing 718A>T was obtained using the primer B718. In the JG goat population, the 718A>T locus had three genotypes, namely AA, AT, and TT (Fig. 4a), and sequences of the homozygous genotypes are presented in Fig. 4b.

### 3.3 Allele and genotype frequencies of 12298T>C and 718A>T in JG goats

Because six blood genomes could not be successfully amplified using the primer G28a2, the remaining 230 samples were successfully detected for 12298T>C of *Lin28a*, and 236 samples were detected for 718A>T of *Lin28b*. The results regarding allele and genotype frequencies of 12298T>C and 718A>T in JG goats are listed in Table 2. The $\chi^{2}$ test indicated that the genotypic distribution of 12298T>C of the *Lin28a* gene in JG goats was consistent with the Hardy–Weinberg equilibrium ( P>0.05 ). For the 718 locus in *Lin28b*, the frequency of genotypes AA, AT, and TT was 0.466, 0.504, and 0.03, respectively, and the A allele was the dominant allele in JG goats, with an allele frequency of 0.718. The genotypic distribution of 718A>T of *Lin28b* deviated from the Hardy–Weinberg equilibrium ( P<0.01 ).

### 3.4 Influence of fixed effects on litter size in JG goats

Litter size in JG goats was significantly influenced by sire ( P<0.05 ) and parity ( P<0.05 ). The least-squares mean and standard error for the litter size of different genotypes in JG goats are given in Table 3. For the 12298 locus in *Lin28a*, no significant difference was found in terms of litter size between the three genotypes in JG goats ( P>0.05 ).

**Table 3:** Least-squares mean and standard error for litter size of different genotypes of 12298T>C of *Lin28a* and 718A>T of *Lin28b* in Jining Grey goats.

Locus	Genotype	Numbers	Litter size
12298T>C	TT	86	$2.35^{a}\pm 0.43$
(*Lin28a*)	TC	102	$2.36^{a}\pm 0.52$
	CC	42	$2.42^{a}\pm 0.45$
718A>T	AA	119	$2.10^{b}\pm 0.10$
(Lin28b)	AT	110	$2.58^{a}\pm 0.11$
	TT	7	$2.93^{a}\pm 0.16$

Least-squares means with the same superscript for the same locus have no significant difference ( P>0.05 ). Least-squares means with different superscripts for the same locus differ significantly ( P<0.01 ).

For the 718 locus of *Lin28b*, the JG goat does with genotypes TT and AT had 0.83 ( P<0.01 ) or 0.48 ( P<0.05 ) more kids, respectively, than those with genotype AA. No significant difference in terms of litter size was found between TT and AT genotypes in JG goats ( P>0.05 ). These results preliminarily revealed an association between allele T of the 718 locus in the *Lin28b* gene and large litter size in JG goats.

## 4 Discussion

In this study, the Lin28a protein distribution in goat ovaries was detected by means of the IHC method. Unfortunately, the Lin28b protein expression was not conducted by IHC due to a lack of suitable antibodies. A mutation in the *Lin28a* and *Lin28b* genes was found and detected in JG goats, respectively.

*Lin28a* and *Lin28b* were both involved in modulating follicle development. *Lin28a* and *Lin28b* transcripts or proteins were observed in mouse and human fetal and adult ovaries (Childs et al., 2012; El-Khairi et al., 2012; Grieco et al., 2013; Flemr et al., 2014; Mamsen et al., 2017), the ovaries of *Drosophila melanogaster* (Stratoulias et al., 2014), and some ovarian cancers (Enriquez et al., 2015; Hsu et al., 2015). Most research should focus on the roles of the *Lin28a* or *Lin28b* gene in the maintenance of the undifferentiated PGCs. *Lin28a* expression increased in the developing human ovary between 6 and 9 weeks post-conception, but *Lin28b* was expressed at lower levels and did not change with development (El-Khairi et al., 2012; Mamsen et al., 2017). Childs et al. (2012) reported that *Lin28a* was expressed highly at 8–11 weeks of gestational age (wga) (PGC stage) and decreased significantly between 8–11 and 14–16 wga and remained low at 17–20 wga. The expression of precursor pri-*let-7* mirrored that of the *Lin28a* gene, and the expression of *Lin28b* was unchanged (Childs et al., 2012). These results suggest that the high levels of *Lin28a* in undifferentiated germ cells may be required to inhibit the high levels of pri-*let-7* transcripts from being processed into mature miRNAs and to maintain the germline stem cell state. *Lin28a* negatively regulates the expression of oocyte-specific homeobox genes in mouse embryonic stem cells, and the disruption of *Lin28a* affects germ cell development (Park et al., 2012; El-Khairi et al., 2012). So, *Lin28a* may regulate both the maintenance of the undifferentiated PGCs and the earlier steps of oogenesis, following commitment to differentiation and the loss of “stemness” in PGCs (Rosario et al., 2017). The ovary weight of *Lin28a*-knocked-out (KO) mice was significantly lower than that of wild-type mice, and *Lin28a*-KO ovaries harbored reduced numbers of primordial ovarian follicles and live oocytes and resulted in lower total numbers of pups and smaller litter sizes (Shinoda et al., 2013). These findings suggest that *Lin28a* has a role in maintaining the germ cell pool size at embryonic and adult stages.

In the present study, we found that Lin28a protein was expressed in goat follicles at various stages of development. Specifically, in pre-antral follicles, Lin28a was predominantly detected in granulosa cells and oocytes. In antral follicles, the presence of Lin28a was identified in thecal cells, mural granulosa cells, cumulus granulosa cells, and oocytes. These results are consistent with those observed in the sheep ovary (Zhang et al., 2023b). In sheep, Lin28a localized in oocytes, cumulus cells, follicular granulosa cells of antral follicles, and primary follicles in the ovarian cortex (Zhang et al., 2023b). Zhang et al. (2022) reported that *let-7b* contributed to the involution of atretic follicles and corpus luteum in sheep ovaries (Zhang et al., 2022). We found that the expression of *let-7b* and *let-7g* increased in proestrus ovaries and had the highest expression level in the meta-estrus ovaries and then decreased in the diestrus ovaries (unpublished data, Fig. S2 in the Supplement). *Lin28a* and *Lin28b* disturb the maturation process of *let-7* miRNA and are the target of *let-7* miRNA (Graf et al., 2013; Ustianenko et al., 2018; Cotino-Nájera et al., 2024). So, we predicted that *Lin28a* could regulate some biological processes of follicle development through *let-7b* and *let-7g* or partially through other members of *let-7* miRNA. However, this speculation needs further functional verification. Although we find that the Lin28a protein expression level rises with the development of follicles in the IHC figures, it is better to use immunofluorescence staining for more supportive results. Despite its limitations, this study shows the importance of *Lin28a* in follicle development. To comprehensively elucidate the function of Lin28a in goat follicle development, it is essential to manipulate the expression levels of Lin28a in goat follicular granulosa cells and oocytes.

There are few reports on the association between *Lin28a* or *Lin28b* and goat litter size. In this study, no base variation was found in exon 2 and exon 3, while a mutation (12298T>C) in intron 2 of *Lin28a* was found by direct sequencing. The 12298T>C locus lies 73 nucleotides upstream (in the 5'-direction) of the 3' splice site and near the branch site of intron splicing (Fig. S1). The association analysis suggests no significant effect on the litter size of JG goats.

The 718A>T locus in the *Lin28b* gene may affect the litter size of JG goats; the female goats with the T allele have a larger litter size. Unfortunately, the detection of Lin28b protein in goat ovaries was not conducted, and so the reason why 718A>T in *Lin28b* affects litter size in goats needs more studies. *Lin28b* was expressed in the sexual goat hypothalamus, the nodal regulatory center of reproductive functioning (Xie et al., 2025). *Lin28b* may affect the secretion of reproductive hormones or the expression of other genes affecting litter size, which needs more comprehensive research. Even so, the 718 locus of *Lin28b* could be considered to be a candidate locus in goat breeding to improve reproductive performance.

## 5 Conclusion

The present study provided an expression pattern of Lin28a protein in goat ovaries. Lin28a was expressed in the primordial follicles in the cortical stroma of ovaries, granulosa cells, and oocytes at different stages of development and changed with the formation and regression of corpus luteum. No mutation was found in exon 2 and exon 3 of *Lin28a*. A mutation (718A>T) in exon 4 of *Lin28b* may affect the litter size in JG goats. These results provide further evidence that *Lin28a* and *Lin28b* are key regulators of reproduction and that further studies on them are needed.

## Supplement

10.5194/aab-68-435-2025-supplement The supplement related to this article is available online at https://doi.org/10.5194/aab-68-435-2025-supplement.

## Data Availability

The data used and analyzed during this study are available from the corresponding author upon request.
