# Supplementary material for: The expression of Lin28a in the ovaries and the association of Lin28a and Lin28b with litter size in goats
Source: Arch Anim Breed. 2025 Jul 1;68(2):435–43. doi: 10.5194/aab-68-435-2025 (PMC13384392; doi:10.5194/aab-68-435-2025)
Supplement: The supplement related to this article is available online at https://doi.org/10.5194/aab-68-435-2025-supplement. [file aab-68-435-2025-supplement.zip › aab-68-435-2025-supplement-title-page.pdf]

Supplement of Arch. Anim. Breed., 68, 435–443, 2025
https://doi.org/10.5194/aab-68-435-2025-supplement
© Author(s) 2025. CC BY 4.0 License.

Open Access
Archives Animal Breeding

Supplement of

The expression of Lin28a in the ovaries and the association of *Lin28a* and *Lin28b* with litter size in goats

Huili Liang et al.

Correspondence to: Mingxing Chu (mxchu@263.net) and Guiling Cao (cglling@126.com)

- aab-68-435-2025-supplement-title-page.pdf
- Figure S1 and S2 —aab-2024-52.docx
- Supplementary sequences—aab-2024-52.docx

The copyright of individual parts of the supplement might differ from the article licence.
