# Supplementary material for: The expression of Lin28a in the ovaries and the association of Lin28a and Lin28b with litter size in goats
Source: Arch Anim Breed. 2025 Jul 1;68(2):435–43. doi: 10.5194/aab-68-435-2025 (PMC13384392; doi:10.5194/aab-68-435-2025)
Supplement: The supplement related to this article is available online at https://doi.org/10.5194/aab-68-435-2025-supplement. [file aab-68-435-2025-supplement.zip › Supplementary sequences---aab-2024-52.docx]

Supplementary sequence 1 The PCR sequence using primer G28a1

1 TGAAAGAGGA AGTGGTGAAG TGTCCCCCAG GCTAGCCAAA GGACTCCCAA GGTCTGTGCG

61 GCCTCCTGCT TCTGGGGGCC ATCTCTGGAG CCAGGAACCC TGGTGTCCCA GTGGCGAGCA

121 AGCTGAAGGT CCGGGGGCGG GGGGAGAGAA GAGGAGGGGC CTGTCTGGCC GCATGTCCCC

181 GGGCAACGTG TTGTGGAAAG AAAAGCAAAA CTTTCGGGGC ACCGCTGGCC TCAGTTTAAG

241 CTGGGGGAGC GCGGGAGGCG GCCGGGACAG GTATTTTCTC GCCGGCTGCT CGGTAGCTGC

301 AGCCTCCTGG TTGTCCAGCC TCGGGCTGGA CCCTCGGGTC TCCTTGCCGG CTCTCTGATG

361 GGGGTCCTCC AATTCCGTGG CCCCCCAGCT GAGCGCCTTC CCAATCTCCA GGTGGCTGCG

421 CTAAGGCGCC GGAGGAGGCG CCGGAGGAGG CGCCGGAGGA CGCGGCCCGC GCGGCGGAGG

481 AGCCGCAGCT GCTGCACGGT GCCGGCATCT GTAAGTGGTT CAACGTGCGC ATGGGGTTCG

541 GCTTCCTGTC CATGACCGCC CGCGCAGGGG TCGCGCTCGA CCCCCCGGTG GATGTCTTTG

601 TGCACCAGGT GAGAGCGATT TTGGTAACTT TGCCGCAGGA ACGGGATGTT GGCGCCCCTA

661 TCCGAGGGTG GGCGGACGGA CTCGGCTGTT GGGGATTT

Supplementary sequence 2 The PCR sequence using primer G28a2

1 GGCACTGTCT TACTTCGTAG GGGTTTGTCT TTCATGACTG ACATCTTTTA AGTGTATAGG

61 CCTGTTCCTT TATATTTTAC AATTTTACCT TGGTTGATTT CTTATAAATG CTATGTTTAC

121 CAATTTTTTT CTGCTGTTCT AGTTTATTGA GAACCTTCAA TGAGAATAGC TGCAAAAAAT

181 GCTATGGCAT CTGTTTACAT AGTGATTGTT CCTAATGGAA CATAGATTTC TTGTTCTTAA

241 TGGTGGTGAC TCTTCCAATC ACAGTGTCTT TACCAATAAA ATGGAAACGT TGCACCCTTA

301 CTCTTCCAAC CTTAGAGGAA CA*C*GTGTATG CAGGTTTTGA TGTTTGGGGT GTAATAATGA

361 AGACTAACAT TAAATTCTAC CATGCTTCTC TCCCCCAGAG TAAGCTGCAC ATGGAGGGCT

421 TCCGGAGCCT GAAGGAGGGG GAGGCTGTGG AGTTCACCTT TAAGAAGTCC GCCAAGGGCC

481 TGGAATCTAT CCGAGTCACC GGCCCTGGTG GGGTGTTCTG TATTGGGAGT GAAAGGCGGC

541 CCAAAGGGAA GAATGTGCAG AAACGCAGAT CAAAGGGAGA CAGGTATGGA CTGGAAGGCA

601 GCTTCTGTGG GTTGGTAGGG ATGATGAGCA CTCTTCCTTT CTTGGTTGGA CCAGATGCTA

661 AAGATAAAGT GAAGCCTGTG GCCCCTGGCA ACATCTGGAT GTGGGAGGCA AAGAGAGGGT

721 GGTATGTATG

Supplementary sequence 3 The PCR sequence using primer B28E4

1 GAAGTACTTC CTTGCCTTAT GTGAGGAGTG TAAGCTTATT TAAATTATGT AGACAAATCA

61 AAGTGGCATT GCTTAATTTT TAGCAGGTAT AATAAGCAGG TTAACAGTAA AAATGCAAAA

121 TGTAATAAGT CACTTTGAAA ATTCAAACCA AAGTTCCTTG ACCTTATTAG AAATAGGAAA

181 TTATGGACTT GAGAATTGGA CATTCCCTGT TTACATATAA AAAGTTCAGA GCTGAGATCA

241 CGATTTAAAA AAAGAAACAT TTTATAACTG TGGACCTTAT GGGTGCAATT TGAAATCACT

301 TTTCGGCATC TCACCAGACT GAACTAAGCA CATACCAAAC CTATCTTTGA TGAAAAGTTG

361 GGGTTTATTT TTTATATAAG AATATTATCA CTATTACATA AACATACTCA GGACAAAGAA

421 CTTCACTCAG GGAACATACC GTATAATAAT TGTTATTTCT TTACAGAATA GTCTACAGTC

481 CTGCTTACTC AAAACAAACC AAATAACTTA TACCTTTATG TAAGTATTAT GTACTGATGT

541 TAGTAATAAC TACCTCTGAG TTTGACATAG ATCAAAATGT CCAAATATCA ATTATCAGCT

601 CTCCTTTTTT AATTTCATGT GAAAATTACT CAAGGAGACT TCCTCCACTC CTTTCAGATG

661 TGAATATCAC TGACATAAGC ACAGTGTCCA TTTACATGGG TGAAATATTC TGTTTAC*A*GC

721 AAAAGGCTAC CTCATAGTTG ATGCATAGC

Supplementary sequence 4 The PCR sequence using primer B718

1 GTCAGCTCTC CTTTTTTAAT TTCATGTGAA AATTACTCAA GGAGACTTCC TCCACTCCTT

61 TCAGATGTGA ATATCACTGA CATAAGCACA GTGTCCATTT ACATGGGTGA AATATTCTGT

121 TTAC*A*GCAGA AGGCTACCTC ATAGTT
