# Supplementary material for: The expression of Lin28a in the ovaries and the association of Lin28a and Lin28b with litter size in goats
Source: Arch Anim Breed. 2025 Jul 1;68(2):435–43. doi: 10.5194/aab-68-435-2025 (PMC13384392; doi:10.5194/aab-68-435-2025)
Supplement: The supplement related to this article is available online at https://doi.org/10.5194/aab-68-435-2025-supplement. [file aab-68-435-2025-supplement.zip › Figure S1 and S2 ---aab-2024-52.docx]

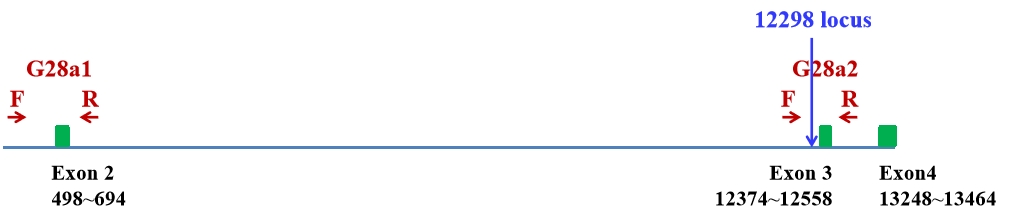


Figure S1. The primer location in the goat *Lin28a* gene (NC_030809).


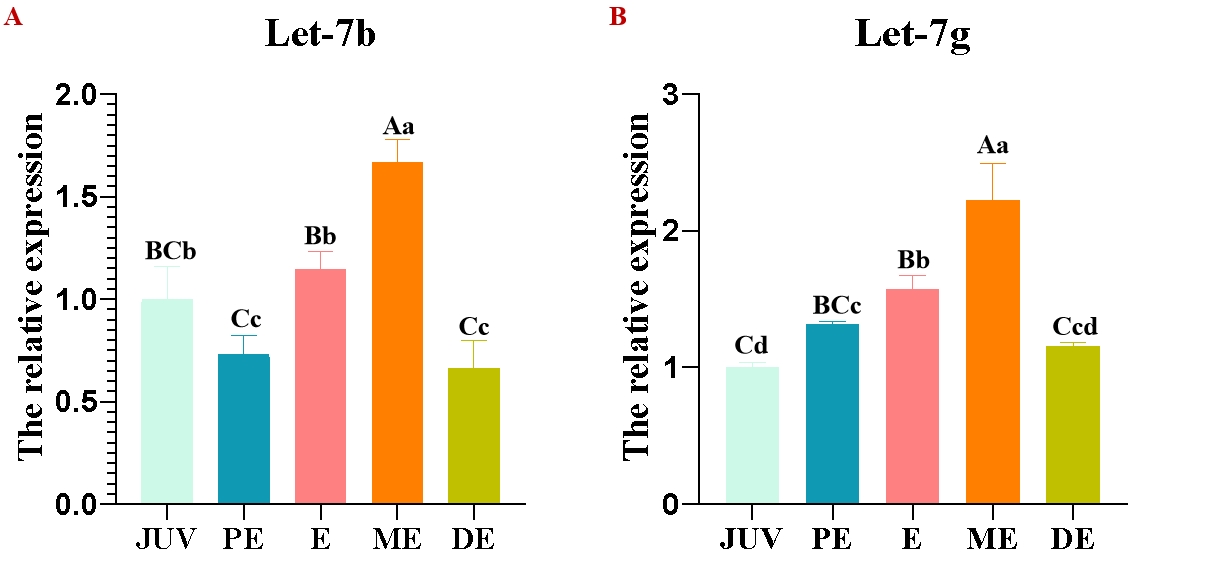


Figure S2. The expression of *let-7b* and *let-7g* in goat ovary tissue. A, *let-7b* and B, *let-7g*. The different capital and small letter means differ significantly (*P* < 0.01 and *P* < 0.05, respectively) in multiple comparisons. JUV, ovaries from one month-old goats. PE, proestrus goat ovaries. E, estrus goat ovaries. ME, metaestrus goat ovaries. And DE, diestrus goat ovaries.
